# Systematic Phosphoproteomic Analysis of Co-Differentially Associated Phosphosites Within the WNK1 C-Terminal Disordered Region

**DOI:** 10.3390/biotech15040095

**Published:** 2026-10-19

**Authors:** Noreen A Khan, Amal Fahma, Athira Perunelly Gopalakrishnan, Alimath Sambreena, Rajesh Raju, Athira C. Rajeev

**Affiliations:** Centre for Integrative Omics Data Science (CIODS), Yenepoya (Deemed to be University), Mangalore 575018, Karnataka, India; noreen.a678@gmail.com (N.A.K.); amalfahma7@gmail.com (A.F.); athirajrf@yenepoya.edu.in (A.P.G.); alimathsambreena.ciods@yenepoya.edu.in (A.S.)

**Keywords:** WNK1 kinase, phosphoproteomics, co-differential association, intrinsically disordered region

## Abstract

With-No-Lysine Kinase 1 (WNK1) is an atypical serine/threonine kinase involved in ion homeostasis, blood pressure regulation, migration and proliferation. Its intrinsically disordered regions (IDRs) support dynamic phosphorylation patterns. Here, we compiled 895 human phosphoproteomic profiling datasets and 352 differential phosphoproteomic datasets to systematically characterise the phosphorylation landscape associated with WNK1. Among reported WNK1 phosphosites, S2027, S2029, and S2032, located within an IDR, were the most frequently detected. Using a one-sided Fisher’s Exact Test followed by three sequential bias-correction filters, we identified 387 phosphosites in other proteins (PsOPs) showing positive co-differential association and 34 showing negative co-differential association with these three WNK1 sites. Computational approaches and database integration identified two candidate upstream kinases (EIF2AK1 and MAPK3), eight binary interactors, and 26 candidate substrate proteins. GO enrichment revealed site-specific functional categories: S2027-associated proteins were enriched in Ras/small GTPase signaling, chromatin organization, and cell-cycle-related processes; S2029-associated proteins in chromosome organization and transcriptional regulation; and S2032-associated proteins mainly in GTPase-related molecular functions. These findings provide a phosphosite-centric resource and computationally supported candidate associations that generate testable hypotheses for further experimental investigation of WNK1-associated signaling.

## 1. Introduction

With-No-Lysine Kinase 1 (WNK1, also referred to as PRKWNK1) is an atypical serine/threonine protein kinase distinguished by its unconventional catalytic domain [1]. WNK1 was the first member to be identified in the WNK kinase family, which also encompasses WNK2, WNK3, and WNK4, recognised due to their sequence similarities and shared structural characteristics following the original identification of WNK1 [2]. These kinases collectively play a role in regulating ion balance, cellular volume, and various physiological processes, particularly within the renal and nervous systems [3,4,5].

The WNK1 gene undergoes alternative splicing, resulting in two primary isoforms: the full-length long isoform (L-WNK1) and the kidney-specific WNK1 (KS-WNK1) [6]. KS-WNK1, which lacks the kinase domain, modulates the function of L-WNK1, especially in the distal nephron [7]. WNK1 is mainly found in the cytoplasm, although research indicates it can dynamically redistribute in response to signaling cues and may interact with membrane-associated proteins [8]. It is encoded on chromosome 12p13.3, with its full-length isoform comprising 2382 amino acids and having a molecular weight of about 250 kDa.

WNK1 is characterised by a distinctive kinase domain, autoinhibitory regions, coiled-coil structures, and areas that facilitate interactions with downstream effectors such as SPAK (STE20/SPS1-related proline/alanine-rich kinase) and OSR1 (oxidative stress-responsive kinase 1) [9]. These features enable its involvement in signal transduction pathways associated with electrolyte transport [10]. Its primary role is to ensure electrolyte balance by regulating Na^+^, K^+^, and Cl^−^ transporters in epithelial cells [11]. The activity of WNK1 is influenced by post-translational modifications, particularly phosphorylation at Thr-60 by AKT/PKB in response to insulin and IGF1 signaling, which integrates it into the PI3K/AKT pathway [12,13,14]. Although this modification does not directly impact the kinase activity of WNK1, it affects its protein interactions and subcellular dynamics, especially during osmotic stress and growth factor signaling [15,16].

Biologically, WNK1 is crucial for regulating blood pressure, renal sodium reabsorption, potassium management, cell volume control, and angiogenesis [17,18,19]. Mutations or overexpression of WNK1 can lead to PHAII, also known as Gordon syndrome, or familial hyperkaliaemic hypertension, a condition characterised by hyperkalaemia and hypertension due to increased NaCl reabsorption in the distal nephron [20,21]. Beyond its role in renal function, WNK1 is also involved in immune cell migration, neurodevelopment, and tumour progression [22,23]. Downstream, WNK1 activates STE20/SPS1-related proline/alanine-rich kinase/oxidative stress-responsive kinase 1 (SPAK/OSR1) kinases, which subsequently phosphorylate NCC (Na^+^-Cl^−^ cotransporter) and K^+^/Cl^−^ cotransporters, thereby fine-tuning electrolyte movement [24]. This pathway is being explored for hypertension treatment, including WNK-SPAK inhibitors such as STOCK1S-50699, which have demonstrated promising antihypertensive effects in animal studies [25]. Additionally, WNK1 is being investigated in oncology as a target to influence cancer cell migration and invasion [26].

Despite this extensive body of work on WNK1, most of the studies have focused on a small number of established regulatory sites within or near the kinase domain. In contrast, unbiased phosphoproteomic studies have repeatedly identified additional WNK1 phosphorylation sites, including several sites within its C-terminal intrinsically disordered region, but these sites have not been systematically integrated across studies. Consequently, the reproducibility of non-canonical WNK1 phosphosites and their potential co-regulation with phosphorylation events elsewhere in the proteome remain largely unexplored. This represents a specific knowledge gap that can be addressed through cross-study integration of publicly available phosphoproteomic data, enabling recurrent phosphosite prioritization and multi-study assessment of co-differential association patterns.

In our curated phosphoproteomic analysis, S2027, S2029, and S2032 were among the frequently detected WNK1 phosphosites, all located within the C-terminal intrinsically disordered region (IDR; residues 1994–2069), yet their specific regulatory associations and signaling context remain uncharacterised. This study therefore performed a systematic analysis of co-differential associations, candidate interactors, kinases and substrates linked to these three phosphosites. This work provides, to our knowledge, the first cross-study assessment of this understudied region of WNK1 and a hypothesis-generating resource for future targeted validation.

## 2. Materials and Methods

### 2.1. Compilation and Analysis of Global Phosphoproteomics Data for WNK1 Phosphosites

A systematic literature search was conducted in PubMed using the query “phosphoproteomics” OR “phosphoproteome” NOT “Plant” NOT “Review”, restricted to human cellular and tissue studies. The search, identified >3825 publicly available phosphoproteomics research articles. Titles and abstracts were screened to exclude plant studies, reviews, and non-mass-spectrometry-based studies. Full texts and supplementary datasets were subsequently examined to identify studies reporting at least one Class-1 WNK1 phosphosite. As localization metrics differed among the compiled phosphoproteomic studies, phosphosite localization was assessed using the metric reported by the original study. Where localization probability was available, a localization probability ≥ 75% was used as the acceptance criterion; where localization probability was not reported but an A-score was provided, an A-score ≥ 13 was used. The two metrics were not treated as numerically equivalent; each value was interpreted according to the metric reported by its source study.

For consistency in peptide identification and phosphosite mapping, only datasets generated from LysC- and/or trypsin-digested peptides were included. Datasets using alternative proteases, including chymotrypsin and Glu-C, were excluded because differences in proteolytic specificity may affect peptide coverage and phosphosite detection. Datasets were classified as either differential or profile datasets. Differential datasets included studies reporting quantitative comparisons between a defined experimental condition and its corresponding control, whereas profile datasets treated experimental and control samples as independent phosphosite-detection profiles based on the presence or absence of Class-1 sites, without paired quantitative comparisons. Datasets were further annotated according to the phosphopeptide-enrichment strategy used before mass spectrometry: STY, encompassing combined Ser/Thr/Tyr enrichment methods (e.g., IMAC and TiO_2_); ST, targeting Ser/Thr phosphorylation; and Y, targeting Tyr phosphorylation (e.g., pTyr-1000 or anti-pTyr antibody enrichment). This classification enabled assessment of potential enrichment-associated biases in phosphosite detection.

For quantitative datasets, Class-1 phosphosites were designated as upregulated (fold change ≥ 1.3) or downregulated (fold change ≤ 0.76) based on the differential results reported in the respective studies and their reported statistical criteria (*p* < 0.05). Fold-change values were retained as reported and were not independently used to reclassify the differential datasets. No additional recalculation of fold changes or statistical correction was performed because of heterogeneity among quantification approaches, including label-free, SILAC, and TMT-based methods.

Protein identifiers were standardised to HGNC gene symbols [27], and phosphosite positions were subsequently mapped to canonical UniProt accessions [28] and isoform numbering using an in-house Python-based mapping pipeline (v3.13.0). Where necessary, ±7-residue sequence windows surrounding each reported site were used to resolve positional ambiguities arising from non-canonical isoforms or outdated reference sequences. Biological and experimental conditions, including stimulus, cell line, genetic perturbation, and treatment duration, were manually curated and assigned standardised condition codes as previously described (Sanjeev et al., 2024) [29], enabling consistent cross-study classification and assessment of potential experimental biases.

### 2.2. Identification of Frequently Detected Phosphorylation Sites in WNK1

All Class-1 WNK1 phosphosites identified across the assembled profile and differential datasets were compiled. For each site, two frequency measures were calculated: (i) the number of profile datasets in which the site was detected and (ii) the number of datasets in which it was differentially regulated (upregulated or downregulated according to the criteria in Section 2.3). Sites were ranked independently according to each measure. A phosphosite was considered predominant based on its detection frequency in differential datasets. Top three frequently detected phosphosites were interestingly a part of the same peptide with different localization probabilities. Three phosphosites (S2027, S2029, S2032) met these criteria and were selected for subsequent correlation and network analyses (Section 2.3 and Section 2.4). The next-ranked sites were S185, S2012, and S2011, showing a clear drop in differential frequency beyond the top three. This classification was restricted to recurrently observed Class-1 sites in unbiased shotgun phosphoproteomic datasets. Consequently, sites reported exclusively through targeted approaches, such as phospho-specific antibody assays or site-directed mutagenesis, were not included unless independently detected at the defined Class-1 confidence level within the assembled datasets.

### 2.3. Analysis of Phosphosites in Proteins Showing Co-Differential Associations with Frequently Detected WNK1 Phosphosites

To identify phosphosites in other proteins (PsOPs) that exhibited positive or negative co-differential association with the frequently detected WNK1 phosphosites, each quantitative differential dataset was analysed independently. As the datasets encompassed diverse biological systems, experimental conditions, mass spectrometry platforms, and analytical workflows, reprocessing of the raw data was not feasible. Consequently, co-differential association was determined directly from the differential phosphorylation calls reported in the original studies, following the general analytical framework previously established in our previous studies [30,31,32]. Datasets were organised and analysed according to their quantitative phosphorylation profiles to pinpoint phosphosites in other proteins (PsOPs) that display positive or negative co-differential association patterns relative to the frequently detected WNK1 phosphosites (S2027, S2029, S2032).

For each frequently detected WNK1 phosphosites (designated w), only datasets in which that site was differentially regulated were considered. Co-detected phosphosites in other proteins (designated o) were classified based on their direction of regulation relative to the corresponding WNK1 phosphosite, resulting in four possible categories:

- UoUw: PsOP upregulated when the WNK1 phosphosite was upregulated.
- DoDw: PsOP downregulated when the WNK1 phosphosite was downregulated.
- DoUw: PsOP downregulated when the WNK1 phosphosite was upregulated.
- UoDw: PsOP upregulated when the WNK1 phosphosite was downregulated.

The UoUw and DoDw categories were defined as positively co-differential association events, whereas DoUw and UoDw were classified as negatively co-differential association events relative to the corresponding WNK1 phosphosite.

The statistical significance of each candidate WNK1 phosphosite-PsOP association was evaluated using a one-sided Fisher’s Exact Test (FET) applied to an appropriate contingency table. Candidate PsOPs were retained only if they simultaneously satisfied the following criteria: (i) an FET *p*-value < 0.05; (ii) the magnitude ratio criterion described below; (iii) evidence from at least three distinct experimental conditions, defined by unique experimental condition codes; and (iv) validation across a minimum of three independent studies, distinguished by unique PubMed IDs. Phosphosites fulfilling all four criteria were classified as high-confidence PsOPs and were carried forward for downstream biological interpretation.

To account for multiple testing across the large number of phosphosite pairs evaluated for each WNK1 site, we applied Benjamini–Hochberg FDR correction to the Fisher’s exact test *p*-values. The resulting q-values reflect the minimum false discovery rate at which each association would be significant. This correction encompassed all phosphosite-pair combinations tested per WNK1 frequently detected site. Associations with q < 0.05 were considered significant, and effect sizes were expressed as odds ratios with 95% confidence intervals to quantify the strength of co-differential association.

#### Bias Correction

The unfiltered set of FET-significant candidates was found to be disproportionately influenced by a small number of datasets contributing unusually large numbers of co-regulated phosphosites. To correct for this, three additional confidence filters were applied sequentially.

1. Magnitude ratio filter. For each candidate PsOP, we calculated the ratio of supporting to opposing dataset counts: Σ (nUwUo + nDwDo)/Σ (nUwDo + nDwUo) for candidates tested as positively associated and the inverse ratio for those tested as negatively associated (datasets with a denominator of zero were assigned a ratio of 1 to allow calculation). A PsOP was retained only if this ratio was at least 10% of the total number of differential datasets recorded for the corresponding WNK1 phosphosite.
2. Independent study filter. To prevent a single highly productive study from disproportionately driving a PsOP’s apparent significance, each candidate pair was additionally required to be supported by at least 3 distinct published articles (distinguished by PubMed ID), with more articles supporting the assigned correlation direction than the opposing one.
3. Distinct experimental condition filter. Similarly, each candidate pair was required to be supported by at least 3 distinct experimental condition codes (i.e., biologically or experimentally distinguishable conditions such as different stimuli, cell lines, or genetic perturbations; see condition coding in Section 2.1), again with more conditions supporting the assigned direction than the opposing one.

PsOPs satisfying all four criteria, FET *p* < 0.05, the magnitude ratio threshold, the independent-study threshold, and the distinct-condition threshold, were classified as high-confidence WNK1 phosphosite-PsOP pairs and carried forward for all subsequent biological interpretation (interactor, kinase/substrate, pathway, and gene-set enrichment analysis). Additionally, FDR correction (q < 0.05) was applied to the FET *p*-values to evaluate the robustness of the observed associations.

### 2.4. Acquisition of the WNK1 Protein Interactome and Identification of Upstream Kinases, and Downstream Substrates

Protein–protein interaction partners for WNK1 were retrieved from multiple publicly available databases, including HPRD [33], BIND [34], BioGRID [35], ConsensusPathDB version 35 (accessed on 22 May 2023) [36], CORUM (accessed on 3 March 2023) [37], and RegPhos 2.0 (accessed on 24 May 2023) [38].

Experimentally validated upstream kinases and substrates, along with their associated WNK1 phosphosites, were curated from databases such as PhosphoSitePlus v6.8.0. (accessed on 22 May 2023) [39], Phospho.ELM 9.0 (accessed on 24 May 2023) [40], and RegPhos 2.0 (accessed on 24 May 2023). Additionally, predicted upstream kinases and substrates for WNK1 were identified using in silico prediction tools such as NetworKIN (predicted on 4 January 2023) [38], AKID (predicted on 24 May 2023) [41], and iKiP DB [42] to support kinase–substrate relationship analysis.

Because several of these tools require individual protein sequences as query inputs, the complete set of required RefSeq protein sequences was processed programmatically through API-supported workflows. WNK1 phosphosites identified through Synthetic Peptide Screening by Johnson et al. (2023) [43] and functional site annotation by Ochoa et al. (2020) [44] were incorporated using a 90th-percentile signi9ficance threshold for inclusion.

### 2.5. Functional Enrichment and Data Visualisation Tools

**Figure 1:**
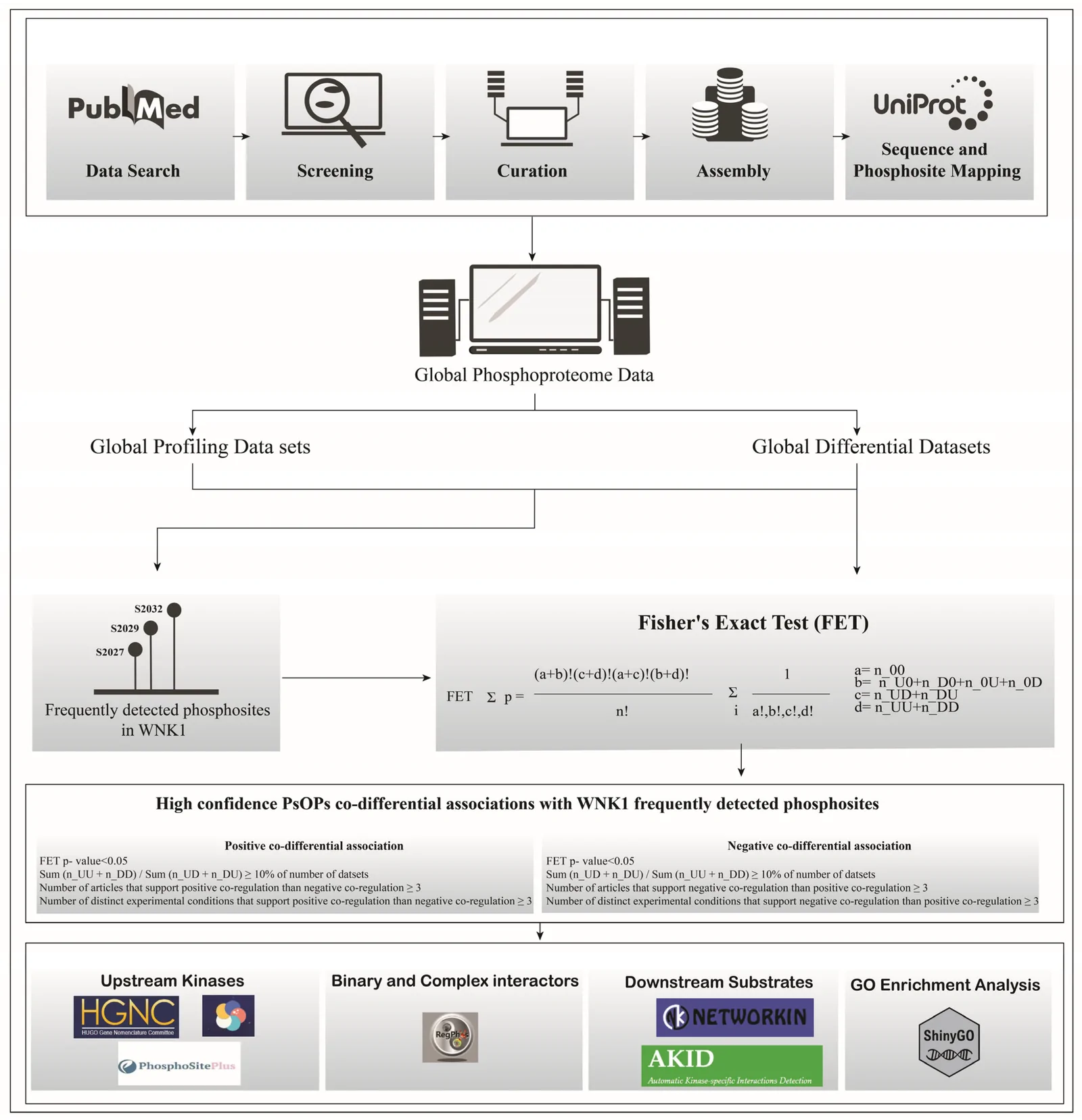
Workflow for the assembly and analysis of WNK1 phosphosites and their co-differential associations with phosphosites in other proteins.

Functional gene-enrichment analysis was performed using the ShinyGO v0.80 (released on 1 May 2024) [45] on PsOPs that exhibited positive co-differential associations with WNK1 frequently detected phosphosites. The lollipop plots were visualised using M2Viz [46]. The intrinsically disordered regions of WNK1 were predicted and visualised using the IUPred3 tool [47]. RAWGraphs [48] (https://www.rawgraphs.io/) was used to generate circular dendrograms of interactors. Cytoscape version 3.10 [49] was used to map the relationships between frequently detected WNK1 phosphosites and PsOPs within kinase-substrate networks. The overall workflow is depicted in Figure 1.

## 3. Results

### 3.1. Data Statistics of WNK1 Phosphoproteome and Identification of Frequently Detected WNK1 Phosphosites Across the Global Phosphoproteome

**Figure 2:**
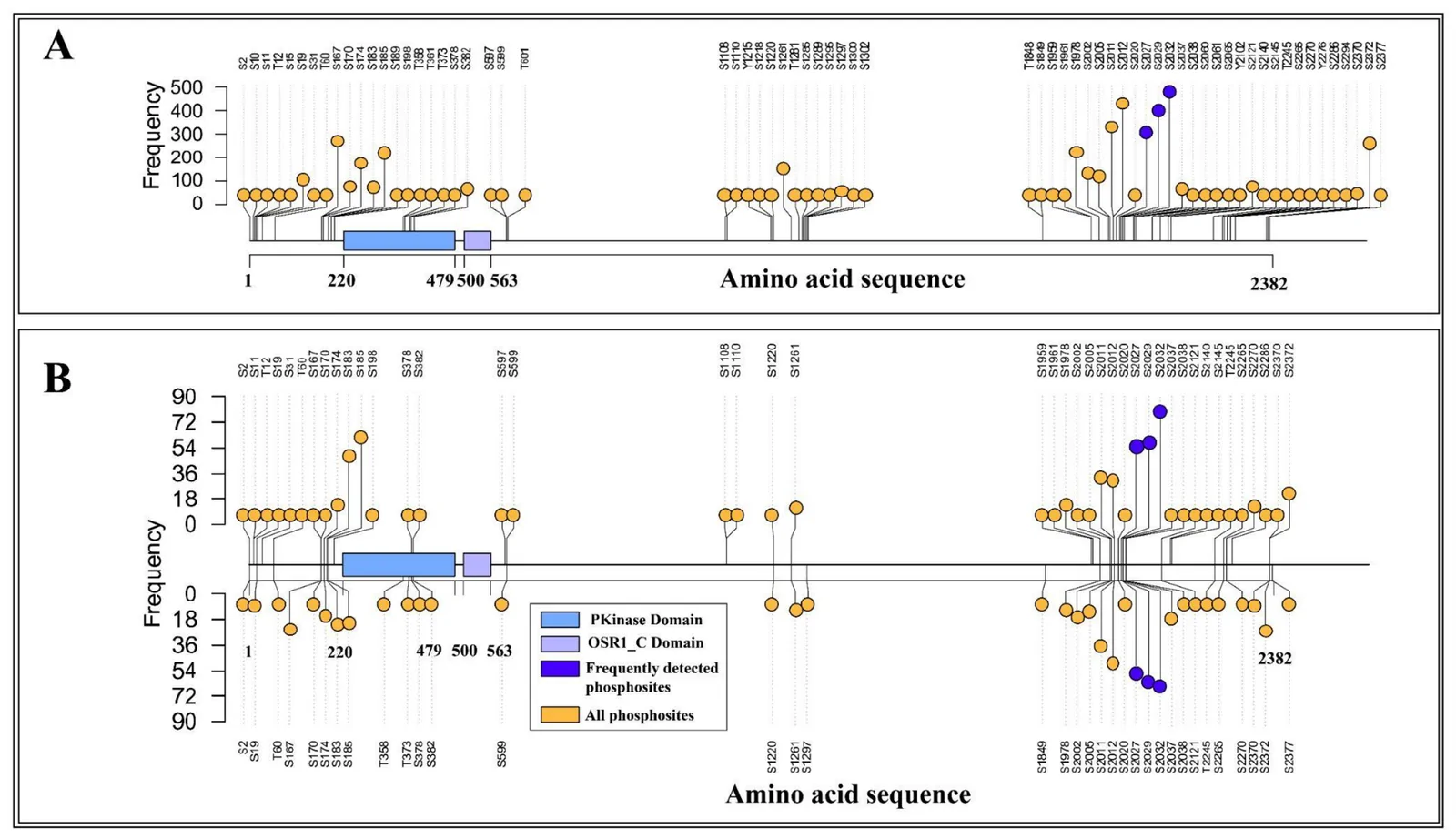
Lollipop plots depicting the frequency of WNK1 phosphosites identified from human cellular profiling and differential phosphoproteomic datasets. The *X*-axis represents the WNK1 amino acid sequence length, and the *Y*-axis represents the number of datasets in which each phosphosite was detected. (**A**) WNK1 phosphosites identified in human cellular profiling datasets. (**B**) WNK1 phosphosites identified in human cellular differential datasets. Frequently detected phosphosites selected for further analysis are highlighted in purple. Functional domains, including the protein kinase (PKinase) domain and OSR1_C domain, are indicated.

After thorough review of more than 3825 publicly available global phosphoproteomic articles from human cellular research, 895 profiling datasets and 352 differential datasets related to WNK1 were identified and chosen based on data quality standards and the presence of Class I phosphosites as listed in Supplementary Tables S1 and S2. These datasets, gathered under various experimental conditions, were considered appropriate for the current analysis. From the profiling datasets, 134 unique WNK1-linked phosphosites were systematically compiled, of which 47 showed measurable differential regulation across the differential datasets (Figure 2A,B). For subsequent analysis, only the phosphosites with the highest detection frequency among these differentially regulated sites were considered, yielding three frequently detected WNK1 phosphosites, S2032, S2029, and S2027, detected in 149/352, 124/352, and 115/352 datasets, respectively (Supplementary Table S3). Because these three phosphosites occur within the same peptide, they were considered related at the peptide level but analysed independently at the individual phosphosite level in subsequent analysis.

**Figure 3:**
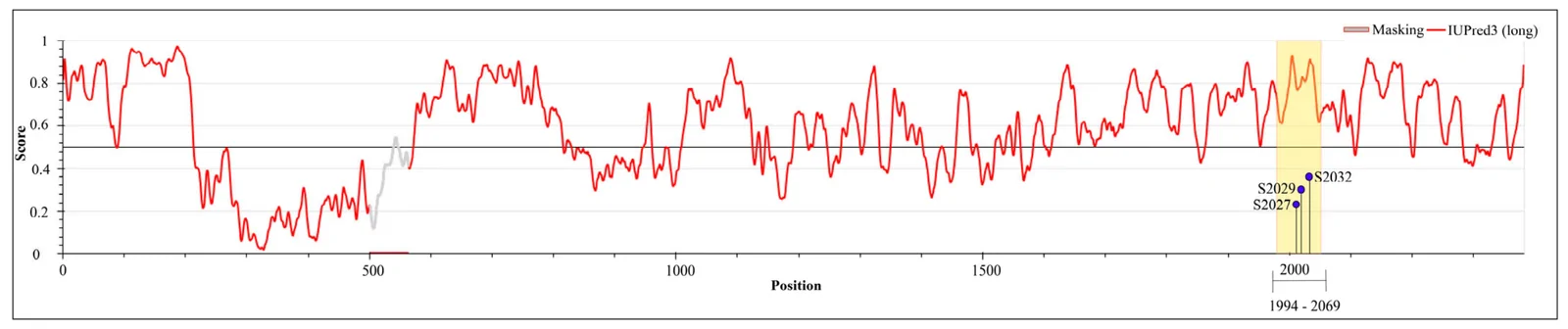
Bokeh plots generated using IUPred3 to predict intrinsically disordered regions (IDRs) in WNK1. The red line shows the disorder score, and the grey regions indicate masked sequences. The yellow-highlighted region covers residues 1994–2069. Within this region, the three serine residues, S2027, S2029, and S2032, are highlighted in purple. The *X*-axis represents amino acid positions, and the *y*-axis shows the disorder score (0–1). Scores above 0.5 indicate regions predicted to be intrinsically disordered, while scores below 0.5 indicate regions predicted to be ordered.

Although the functional role of WNK1 S2032 has not yet been reported, ADP-ribosyl cyclase/cyclic ADP-ribose hydrolase 1 (CD38) has been identified as a regulatory protein that may directly or indirectly influence the phosphorylation status of this site [50]. In addition, in vitro evidence suggests that Serine/threonine-protein kinase mTOR (mTOR) may directly or indirectly regulate phosphorylation at WNK1 S2032 [51]. All frequently detected phosphosites are localised outside the kinase domain, and the kinase is therefore classified as PoKD (predominant phosphosite outside the kinase domain) [52]. Intrinsically disordered regions (IDRs) of WNK1 were predicted using the IUPred3 tool [47] to determine the structural context of the identified frequently detected phosphosites, and these phosphosites were found to reside within the predicted IDRs, as visualized in Figure 3.

### 3.2. High-Confidence PsOPs Showing Co-Differential Associations with WNK1 Frequently Detected Phosphosites

**Figure 4:**
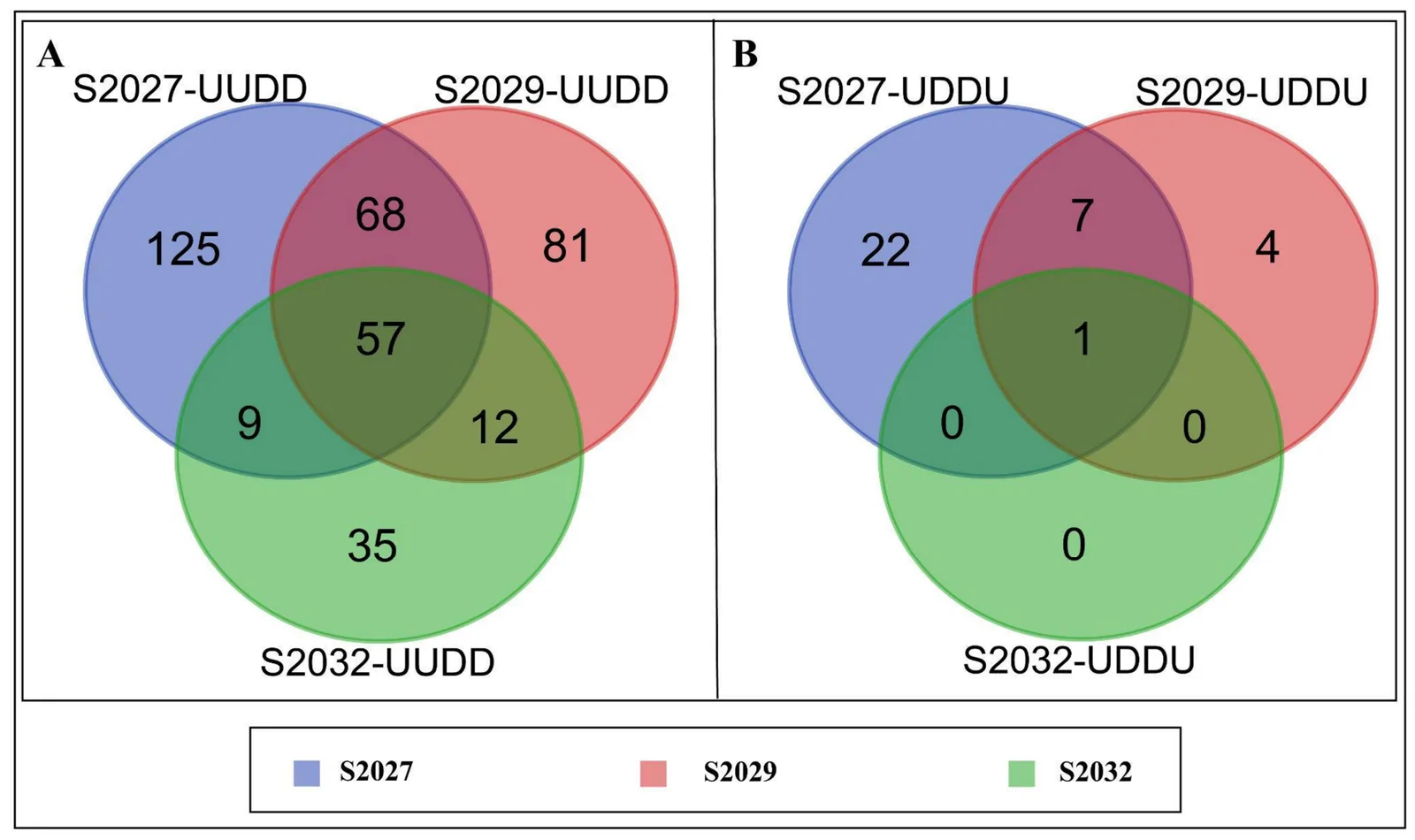
Venn diagrams showing the overlap of phosphosites showing co-differential associations with WNK1 S2027, S2029, and S2032 in the C-terminal disordered region. (**A**) Positively co-differentially associated phosphosites identified by the UUDD configuration. (**B**) Negatively co-differentially associated phosphosites identified by the UDDU configuration. The blue, red, and green circles represent S2027, S2029, and S2032, respectively. The central overlapping region represents phosphosites showing co-differential associations with all three WNK1 phosphosites.

We identified 259 PsOPs showing positive and 30 PsOPs showing negative co-differential associations with WNK1 (S2027), 218 PsOPs showing positive and 12 PsOPs showing negative co-differential associations with WNK1 (S2029), and 113 PsOPs showing positive and 1 PsOP showing negative co-differential associations with WNK1 (S2032) (Supplementary Tables S4–S9). Comparing the associations across the three frequently detected WNK1 phosphosites, Venn analysis identified 387 unique PsOPs with positive co-differential associations and 34 unique PsOPs with negative co-differential associations after accounting for overlapping entries across the three phosphosite-specific lists. Of these, 57 positive and 1 negative PsOP were common to all three WNK1 phosphosites (S2027, S2029, and S2032) and are depicted in Figure 4. Following FDR correction, the number of retained PsOPs remained unchanged from the initial FET-based selection.

To provide functional context for the common PsOPs, representative phosphosites with previously reported site-specific evidence were examined. Among the positive PsOPs, phosphorylation of RIOK2-S483 has been reported to promote late-stage pre-40S ribosomal maturation and support protein synthesis and cell proliferation [53]. DAXX-S737/S739 phosphorylation has been associated with enhanced SUMO1-selective interactions and stress-induced apoptotic responses [54]. Also, calcineurin substrate profiling identified RANBP3 as a potential calcineurin substrate and revealed a conserved LxVP motif adjacent to the human S126 phosphorylation site [55]. Among the negative PsOPs, HTATSF1 was the only common phosphosite identified. HTATSF1 is involved in transcription and pre-mRNA splicing, and its depletion has been shown to alter transcript abundance and exon usage [56].

**Figure 5:**
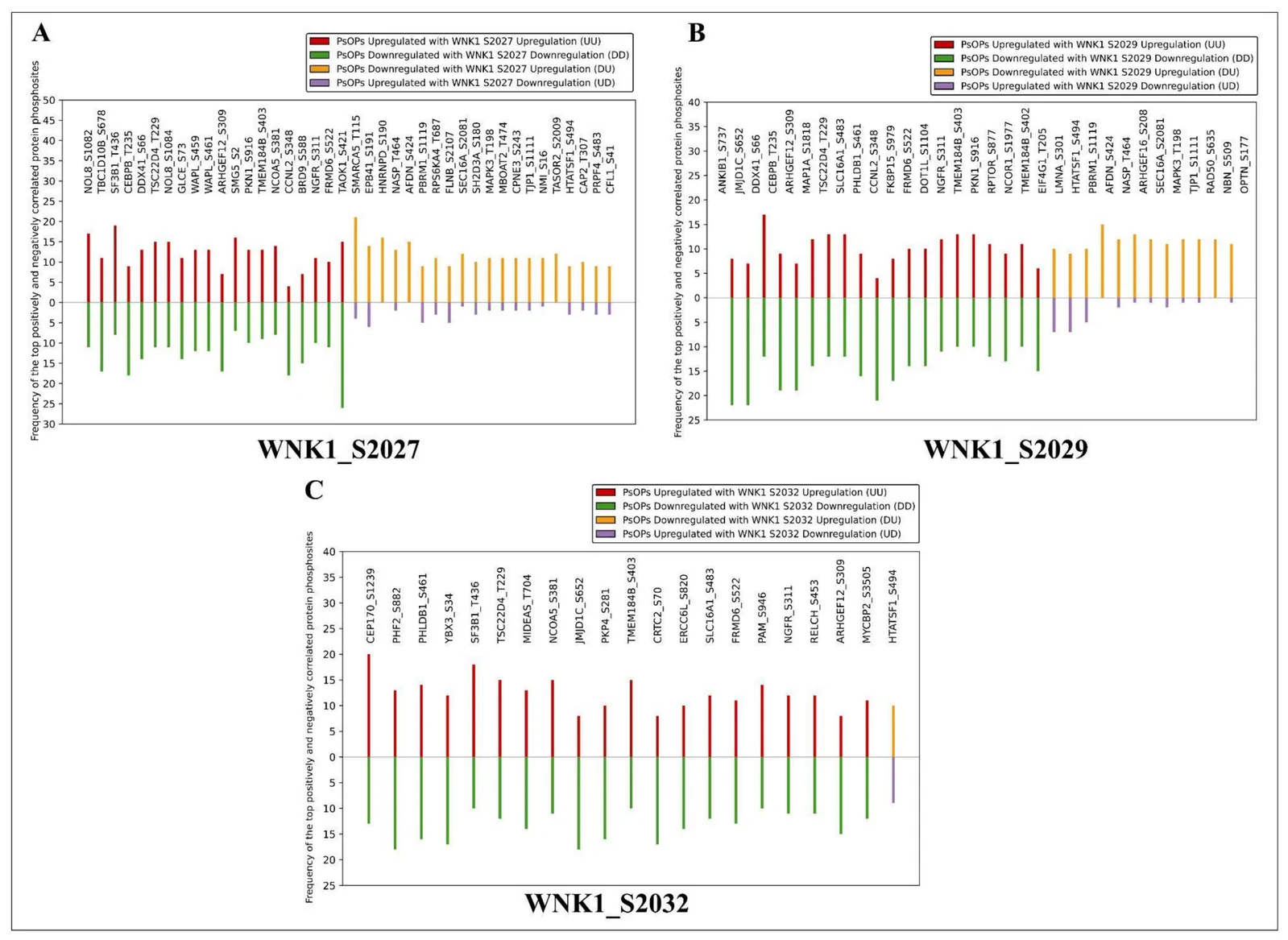
Bar charts illustrating the most frequent positive and negative co-differential associations (PsOPs) with phosphorylation changes at three frequently detected WNK1 C-terminal phosphosites: S2027 (**A**), S2029 (**B**), and S2032 (**C**). The bar charts show the frequencies of PsOPs exhibiting positive or negative co-differential phosphorylation patterns with the three WNK1 phosphosites across the curated differential phosphoproteomic datasets. The colours indicate the type of co-differential associations: red signifies upregulated PsOPs with WNK1 upregulation (UU), green indicates downregulated PsOPs with WNK1 downregulation (DD), orange represents upregulated PsOPs with WNK1 downregulation (DU), and purple denotes downregulated PsOPs with WNK1 upregulation (UD).

Additionally, Figure 5 highlights up to 20 PsOPs showing positive and negative co-differential associations with the frequently detected WNK1 phosphosites. Up to 20 PsOPs were selected separately for each direction. When fewer than 20 PsOPs were available for a given WNK1 phosphosite and direction, all available PsOPs were included. The PsOPs showing the positive and negative co-differential associations with these WNK1 phosphosites are discussed below. The PsOPs showing positive co-differential associations included Nucleolar protein 8 (NOL8) (S1082) with WNK1 (S2027), Ankyrin repeat and IBR domain-containing protein 1 (ANKIB1) (S737) with WNK1 (S2029), Centrosomal protein of 170 kDa (CEP170) (S1239) with WNK1 (S2032), and FERM domain-containing protein 6 (FRMD6) (S522) with WNK1 (S2027, S2029, and S2032). Conversely, HIV Tat-specific factor 1 (HTATSF1) (S494) showed negative co-differential association with WNK1 (S2027, S2029, and S2032).

NOL8, which interacts with beta-catenin to enhance its binding to TCF4 and activate the beta-catenin/TCF signaling pathway, is overexpressed in prostate cancer, promoting cancer cell proliferation, migration, and colony formation [57]. ANKIB1, a member of the RING-between-RING (RBR) E3 ubiquitin ligase family, is implicated in diseases such as inflammation, neurodegeneration, cancer, and metabolic disorders. It regulates MAVS protein levels in human cells in vivo, acting as an immunological regulator to prevent excessive inflammatory responses to cytosolic viral RNA [58]. FRMD6, also known as Willin, serves as an upstream regulator of Hippo signaling, influencing actin cytoskeleton dynamics and neuronal cell mechanical phenotypes via ERK signaling. It plays key roles in neuronal differentiation, myelination, nerve injury repair, vesicle exocytosis, and neurodegeneration-related mechanisms [59]. HTATSF1 is involved in RNA processing and transcription-associated functions and has also been implicated in homologous recombination [60,61].

### 3.3. Binary Interactors Showing Co-Differential Association Among the WNK1 PsOPs

**Figure 6:**
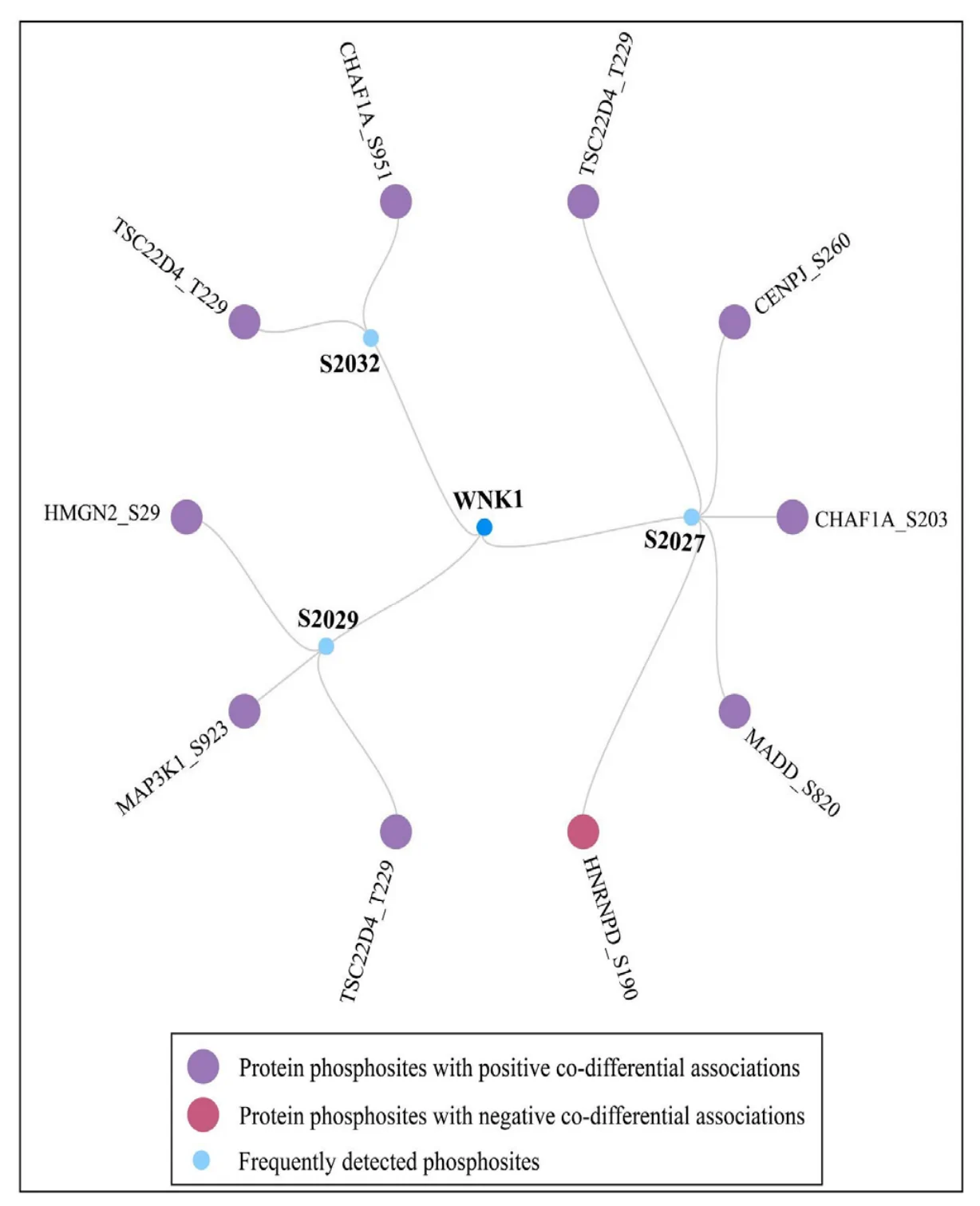
Circular dendrogram representing binary interactors among the PsOPs associated with frequently detected WNK1 phosphosites (S2027, S2029, S2032). Frequently detected phosphosites (S2027, S2029, and S2032) are depicted in purple. The surrounding nodes represent PsOPs identified in the corresponding binary co-differential analysis for each WNK1 phosphosite. Blue circles indicate positive co-differential associations, while pink circles denote negative co-differential associations.

Mapping binary protein-protein interactions is important for gaining insights into the potential biological functions of a protein [62]; we compiled a list of proteins reported to interact with WNK1 from various repositories (Section 2.4). The analysis of the FET results identified 8 PsOPs across 7 proteins that exhibited significant co-differential associations with the frequently detected WNK1 phosphosites S2027, S2029, and S2032 (Supplementary Table S10). On a per-site basis, S2027 was associated with 4 positive (UUDD) and 1 negative (UDDU) phosphosite associations, S2029 with 3 positive (UUDD) and 0 negative (UDDU) associations, and S2032 with 2 positive (UUDD) and 0 negative (UDDU) associations. These associations are presented in Figure 6.

The identified binary interactors include TSC22 domain family protein 4 (TSC22D4) (T229), Centromere protein J (CENPJ) (S260), Chromatin assembly factor 1 subunit A (CHAF1A) (S203; S951), MAP kinase-activating death domain protein (MADD) (S820), Heterogeneous nuclear ribonucleoprotein D0 (HNRNPD) (S190), Mitogen-activated protein kinase kinase kinase 1 (MAP3K1) (S923), and Non-histone chromosomal protein HMG-17 (HMGN2) (S29). The phosphosites mentioned along with the proteins were found to be co-differentially associated in this data.

TSC22D4, involved in protecting cells from hypertonic stress [63], functionally associates with WNK1 in cell volume regulation and osmosensing. Under hyperosmotic stress, TSC22D4, WNK1, and NRBP family members rapidly form biomolecular condensates, a process reliant on IDRs [64]. Exome and genome studies place Autism Spectrum Disorder (ASD) liability on the convergence of many inherited and de novo variants across neurodevelopmental and synaptic networks, and gene pairs that co-occur in affected individuals converge on cytoskeletal and microtubule-related functions, so a co-regulation link between WNK1 and CENPJ is at most a candidate within that framework [65,66]. Both CHAF1A and WNK1 influence cellular responses to ATR inhibition. CHAF1A contributes to DNA replication and replication-stress responses and has been implicated in ATR inhibitor resistance [67], whereas WNK1 regulates signaling pathways and suppresses ATR-mediated cell-cycle arrest [68]. These functions connect both proteins to mechanisms governing cell-cycle progression and DNA damage responses. MADD, which contains a JIP1-type docking motif, is positioned at a critical point in JNK-associated insulin-signaling networks. MADD/IG20 also plays a role in glucose-induced insulin secretion, and its disruption has been linked to hyperglycaemia and type 2 diabetes-related phenotypes, suggesting potential therapeutic relevance [69,70]. HNRNPD interacts with FUS following RMST-induced FUS SUMOylation, and this interaction contributes to the regulation of ATG4D stability and mitophagy in glioblastoma cells [71]. MAP3K1, a protein kinase, regulates critical cellular processes such as growth, differentiation, migration, and survival, playing a key role in signaling pathways that govern cell fate, promoting both survival and apoptosis [72]. HMGN2 was identified as a co-differentially associated partner of WNK1 in our analysis. The biological significance and mechanistic basis of this association remain to be established.

### 3.4. Identification of Potential Upstream Kinases Regulating WNK1 S2027 and S2029

To contextualise the WNK1 phosphosites identified in our analysis, previously reported kinase and regulatory associations were examined using PhosphoSitePlus. WNK1 phosphorylation has been associated with several kinase classes, including AKT1, mTOR, and WNK1 in in vitro studies [51,73,74], while AKT1, MSK1, SGK1, WNK1, WNK2, and WNK3 are annotated as putative in vivo kinases [14,73,74,75,76]. PPP1CB is reported as an upstream phosphatase [77]. Additional regulatory proteins, including AK6, CD38, KLRK1, LRRC8A, and OSR1, have also been associated with specific WNK1 phosphorylation sites [50,73,75,78,79].

**Figure 7:**
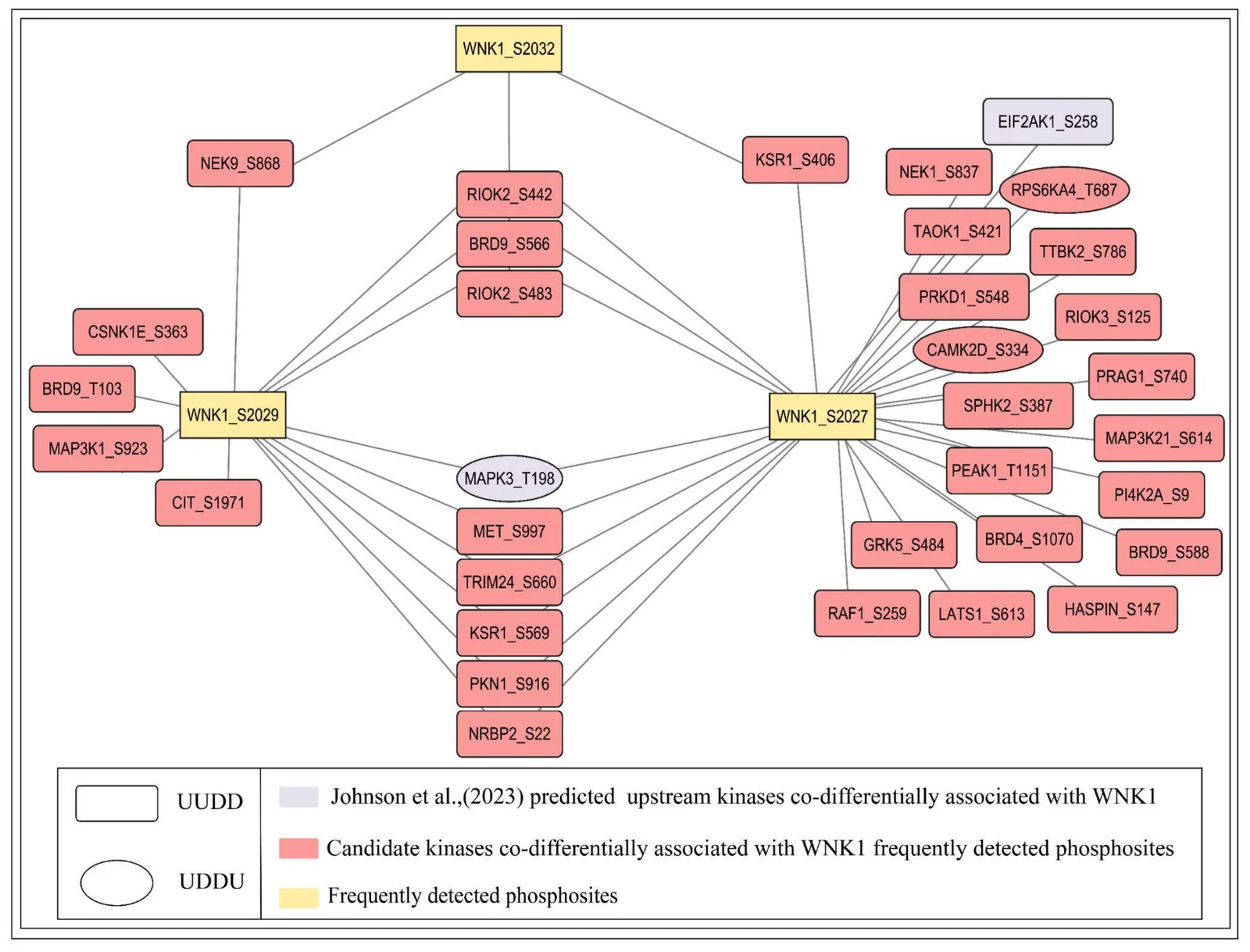
Pictorial representation of kinases identified in association with the frequently detected WNK1 phosphosites (yellow). Kinases identified for the respective WNK1 phosphosites are shown in red, with upstream kinases reported in the study by Johnson et al. (2023) [43] shown in purple. Node shapes indicate the corresponding regulation patterns: rectangles represent UUDD and ovals represent UDDU.

In our analysis, two potential upstream kinases were identified based on kinase-substrate sequence-specificity predictions from the Kinase Library of Johnson et al. (2023) [43]. EIF2AK1 (S258) showed a positive co-differential association with WNK1 S2027 (UUDD), whereas MAPK3 (T198) showed a negative co-differential association with WNK1 S2029 (UDDU) (Figure 7 and Supplementary Table S11). MAPK3 T198 was supported by both the Johnson et al. (2023) [43] prediction and NetworKIN.

EIF2AK1, also known as HRI (Hemin-regulated inhibitor), is a kinase critical to the integrated stress response, regulating protein synthesis under stress conditions [80]. MAPK3, also known as ERK1, is a component of the ERK/MAPK signaling pathway. WNK1 and MAPK3 have previously been reported within an ESR1 and MAPK3-centered expression network in circulating B cells [81]. These results represent candidate kinase–phosphosite associations and do not establish direct phosphorylation or cellular kinase–substrate relationships. These previously reported annotations provide functional context for the candidate kinase associations identified in our analysis, while their relevance to the phosphorylation of the WNK1 sites examined here remains to be experimentally determined.

### 3.5. Additional Kinases Among the PsOPs

Protein kinases are central to cellular signaling, forming a complex and tightly regulated network that governs critical cellular processes, such as cell growth and programmed cell death [82]. In eukaryotes, protein kinases constitute one of the largest gene families, with the human kinome encompassing over 518 protein kinases [83]. Analysis of the FET data identified 34 PsOPs across 30 proteins among the WNK1 PsOPs (Supplementary Table S12). On a per-site basis, S2027 was associated with 26 positive (UUDD) and 3 negative (UDDU) associations, S2029 with 13 positive (UUDD) and 1 negative (UDDU) association, and S2032 with 5 positive (UUDD) and 0 negative (UDDU) associations. These associations are presented in Figure 7.

Among the identified PsOPs kinases are Serine/threonine-protein kinase TAOK1 (TAOK1) (S421), Kinase suppressor of Ras 1 (KSR1) (S569; S406), Serine/threonine-protein kinase N1 (PKN1) (S916), Bromodomain-containing protein 9 (BRD9) (S588; S566), Serine/threonine-protein kinase RIO2 (RIOK2) (S483; S422), Eukaryotic translation initiation factor 2-alpha kinase 1 (EIF2AK1) (S258), Serine/threonine-protein kinase D1 (PRKD1) (S548), Inactive tyrosine-protein kinase PEAK1 (PEAK1) (T1151; S916), Nuclear receptor-binding protein 2 (NRBP2) (S22), Tau-tubulin kinase 2 (TTBK2) (S786), Inactive tyrosine-protein kinase PRAG1 (PRAG1) (S740), Mitogen-activated protein kinase kinase kinase 21 (MAP3K21) (S614), Serine/threonine-protein kinase LATS1 (LATS1) (S613), Hepatocyte growth factor receptor (MET) (S997), and Bromodomain-containing protein 4 (BRD4) (S1070).

Bromodomain-containing protein 4 (BRD4), is a member of the bromodomain- and extra-terminal domain (BET) family of transcriptional regulators [84]. Retinoblastoma (RB), a malignant retinal tumour primarily affecting children [85], shows hyperphosphorylation of BRD4, WNK1, and Cyclin-dependent kinase 1 (CDK1), opening new avenues for RB treatment [86]. KSR1, which amplifies the Raf/MEK/ERK signaling pathway, has been reported to possess protein kinase activity, including serine autophosphorylation and direct phosphorylation of MEK1 [87].

The identification of 34 PsOPs across 30 kinases among the WNK1 PsOPs suggests that WNK1 phosphorylation may be connected to diverse signaling processes, including MAPK signaling, cellular stress responses, growth regulation, and transcriptional control. In particular, the identification of KSR1, BRD4, TAOK1, LATS1, EIF2AK1, and other signaling kinases provides candidate regulatory links that may contribute to the functional signaling context of WNK1. These associations remain predictive and require experimental validation to establish direct kinase–substrate relationships.

### 3.6. Potential WNK1 Substrates Showing Co-Differential Associations

**Figure 8:**
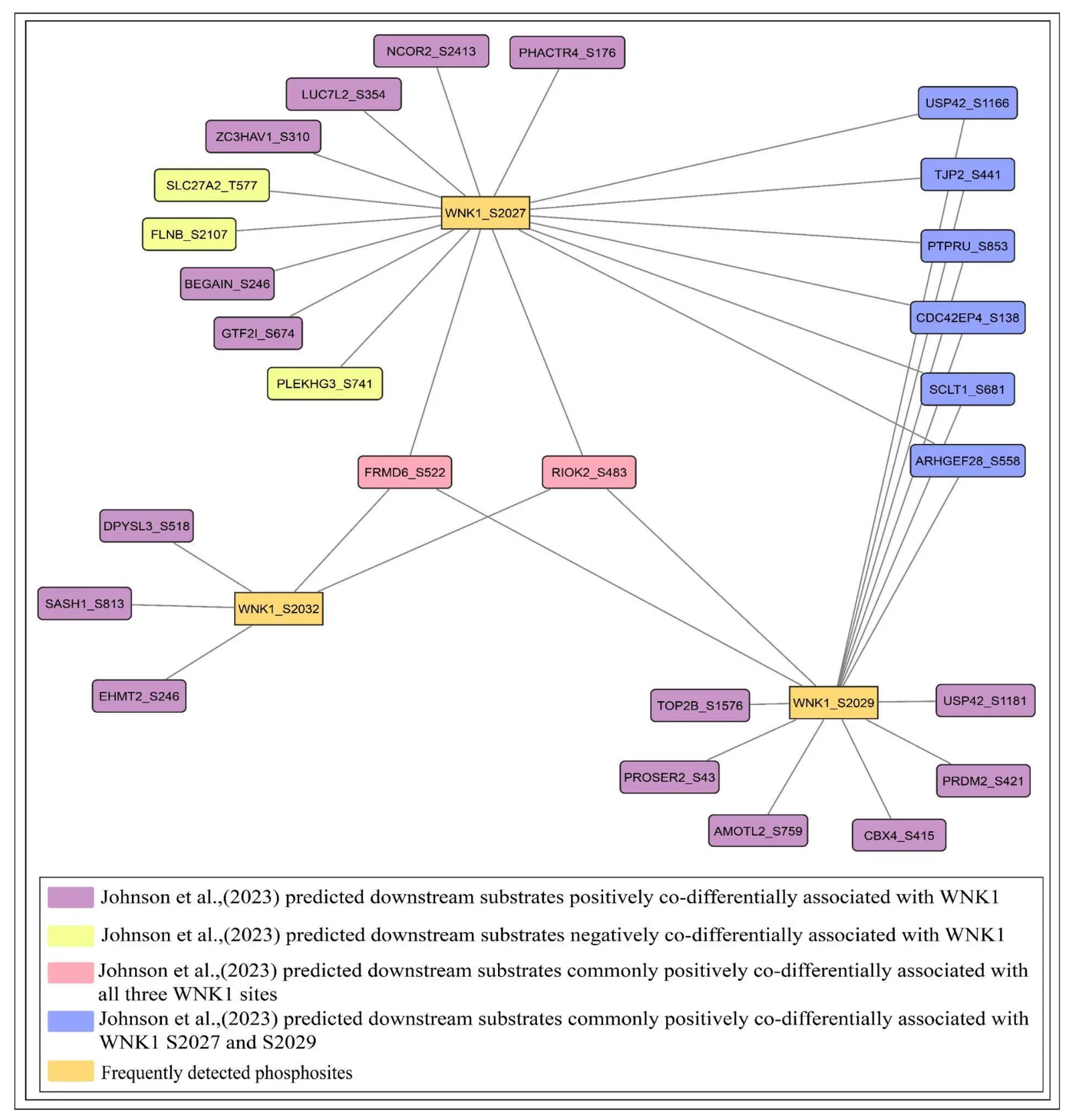
Pictorial representation of potential WNK1 downstream substrates showing co-differential associations corresponding to WNK1 phosphosites S2027, S2029, and S2032. Nodes are distinguished by colour according to the direction of co-differential association. The network includes phosphosites identified in the corresponding analysis for the WNK1 phosphosites [43]. Frequently detected WNK1 phosphosites S2027, S2029, and S2032 are marked in yellow.

For the frequently detected WNK1 phosphosites, S2027, S2029, and S2032, 26 PsOPs across 25 proteins were predicted by Johnson et al. (2023) [43] via their kinase-substrate specificity atlas derived from synthetic peptide library profiling of the human serine/threonine kinome (Figure 8), as listed in Supplementary Table S13. Among these predicted substrates, four were highlighted as key regulators of cell migration and showed positive co-differential associations with frequently detected WNK1 phosphosites. These proteins were receptor-type tyrosine-protein phosphatase U (PTPRU) (S853), dihydropyrimidinase-related protein 3 (DPYSL3) (S518), angiomotin-like protein 2 (AMOTL2) (S759), and SAM and SH3 domain-containing protein 1 (SASH1) (S813), based on their annotated roles in cytoskeletal dynamics and cell motility. Among these predicted substrates, five were highlighted as key regulators of cell migration and showed positive co-differential association with WNK1 frequently detected phosphosites based on their annotated roles in cytoskeletal dynamics and cell motility: receptor-type tyrosine-protein phosphatase U (PTPRU) (S853), Dihydropyrimidinase-related protein 3 (DPYSL3) (S518), Angiomotin-like protein 2 (AMOTL2) (S759), and SAM and SH3 domain-containing protein 1 (SASH1) (S813). This substrate set is inherited directly from an external prediction resource and applied to the three major WNK1 sites; it is a computational prediction, not an independently validated interaction network, and its scope is restricted to the three phosphosites carried forward from the frequency-based site selection.

Among proteins functionally linked to WNK1, PTPRU, a receptor protein tyrosine phosphatase, supports migration and invasion by modulating β-catenin and focal adhesion pathways; in glioma models, reduced PTPRU expression impaired migration and invasion, but β-catenin reactivation restored these effects, underscoring the role of PTPRU in cell movement [88]. DPYSL3 regulates mitosis, migration, and epithelial-to-mesenchymal transition (EMT) in claudin-low breast cancer cells, indicating its role in cytoskeletal dynamics and tumour progression [89]. AMOTL2, a scaffolding protein associated with cell junctions, maintains tight junction integrity and regulates Hippo-YAP/TAZ signaling, coordinating cell polarity and movement in endothelial and epithelial cells during development and vascular remodeling [90]. SASH1, often downregulated in breast cancer, is linked to increased invasion and tumorigenesis via the PI3K-Akt-mTOR signaling pathway, establishing its role as a tumour suppressor in migration regulation [91].

Separately from the network above, the literature also describes a role for WNK1 acting through OXSR1 and SPAK (STK39) to regulate ion cotransporters such as SLC12A2 (NKCC1), driving actin cytoskeleton remodeling critical for leading-edge formation and cell polarity [92]. In breast cancer models, WNK1 promotes migration and invasion by facilitating cytoskeletal rearrangements and extracellular matrix remodeling, contributing to tumour progression [26] Additionally, WNK1 supports metastasis and angiogenesis by modulating vascular permeability and endothelial signaling in cancer [22]. Together, WNK1 and its potential substrates point to a network that integrates ion signaling, actin remodeling, cell–cell junctions, and cell polarity, ensuring effective cell migration across developmental, immune, and cancerous contexts.

### 3.7. Gene Ontology Enrichment Analysis of High-Confidence PsOPs of WNK1

To assess the biological relevance of the identified associations, GO enrichment analysis was performed using ShinyGO v0.80 (released on 1 May 2024) [45]. The input sets comprised 248, 204, and 101 unique proteins positively and negatively co-differentially associated with WNK1 phosphosites S2027, S2029, and S2032, respectively, following application of the 10% FET cutoff. A common background set of 9844 unique proteins, derived from the unfiltered dataset without applying the 10% FET cutoff, was used for all three phosphosites. The complete input and background protein lists are provided in Supplementary Table S14.

For WNK1 S2027, the associated proteins showed enrichment in biological processes related to microtubule depolymerization regulation (~8.76-fold), histone acetylation regulation (~7.39-fold), histone deacetylation regulation (~6.96-fold), Ras protein signal transduction (~2.93-fold), small GTPase-mediated signal transduction (~2.42-fold), chromatin organization (~2.29-fold) and cell cycle (~1.79-fold). Molecular functions were enriched for nuclear thyroid hormone receptor binding (~8.21-fold), nuclear receptor binding (~4.34-fold), and histone binding (~3.03-fold). Cellular components showed enrichment in chromatin (~1.87-fold), nucleoplasm (~1.39-fold), and nuclear lumen (~1.33-fold).

For WNK1 S2029, chromosome organization was enriched ~2.36-fold among the associated proteins. Molecular functions showed enrichment for nuclear receptor binding (~4.88-fold), transcription corepressor activity (~3.47-fold), and transcription coregulator activity (~2.72-fold). Cellular components were enriched in nuclear protein-containing complexes (~1.94-fold), chromatin (~1.87-fold), nucleoplasm (~1.46-fold), and nuclear lumen (~1.43-fold).

For WNK1 S2032, no significantly enriched biological process terms were identified. Molecular functions showed enrichment in GTPase activator activity (~5.43-fold), small GTPase binding (~3.97-fold), GTPase regulator activity (~3.6-fold), and GTPase binding (~3.56-fold). The cellular component terms are condensed chromosomes (~3.76-fold). Full term lists, fold enrichment values, gene counts, and FDR values for all three ontologies are provided in Supplementary Table S15.

## 4. Discussion

This study systematically characterised WNK1 phosphorylation patterns using published phosphoproteomic datasets and identified S2027, S2029, and S2032 within its C-terminal intrinsically disordered region (IDR) as frequently detected phosphosites. These sites were detected across 895 qualitative and 352 quantitative datasets, indicating that phosphorylation within this region is recurrently observed across diverse experimental conditions. The three sites lie outside the canonical WNK1 activation loop and SPAK-docking region and therefore have not, to our knowledge, been characterised in comparable detail elsewhere. Their location within an IDR can also be considered in the broader context of quantitative analysis of disorder hotspots across the human kinome, which provides a framework for assessing the distribution of disordered regions in protein kinases [93]. The clustering of these phosphosites in the C-terminal IDR (residues 1994–2069) is consistent with the broader view that IDRs can respond to changes in cellular physicochemical conditions [94,95].

Several of the candidate associations identified are consistent with previously reported WNK1-related biology. TSC22D4, identified as a binary interactor in our analysis, has been reported to co-assemble WNK1 and NRBP family proteins into IDR-dependent biomolecular condensates under hyperosmotic stress. Similarly, the enrichment of chromatin-associated proteins among the PsOPs and the identification of kinases such as EIF2AK1 (a core integrated stress response kinase) and MAPK3/ERK1 point toward stress- and proliferation-linked regulatory contexts for these sites. Among the predicted substrates, proteins such as PLEKHG3, PTPRU, DPYSL3, AMOTL2, and SASH1 have independently reported roles in cytoskeletal remodeling and cell migration, which is at least directionally consistent with WNK1’s reported involvement in cell motility and cancer cell invasion. We present these as candidate associations requiring experimental validation. Similarly, Similarly, CENPJ is involved in microtubule organization and neuronal development, providing functional context for its association with WNK1 phosphosites [96,97]. These associations provide candidate links between the WNK1 phosphosite patterns to processes involving cytoskeletal organization and neuronal development.

The C-terminal IDR harbouring S2032, S2029 and S2027 is also implicated in WNK1’s capacity to form biomolecular condensates under hyperosmotic stress, a process reported to be driven by the IDR’s sequence properties and modulated by phosphorylation [98,99]. Nguyen et al. (2025) proposed that phosphorylation-dependent charge switching within the WNK1 IDR influences phase separation, based on experiments with phosphorylation-state WNK1 mutants under hypertonic stress [100]. Because S2027, S2029 and S2032 lie within this IDR, they may be relevant to the phosphorylation-dependent regulation of IDR behaviour described in these studies. However, this remains an inference based on the location of the identified sites and previous literature rather than a finding of the present study. Importantly, none of the 352 differential contrasts assembled here involves an osmotic or tonicity perturbation, so the present data cannot test whether these sites respond to the condition under which WNK1 condensation has been characterised. Direct experiments using defined osmotic perturbations and site-specific WNK1 phosphosite mutants would therefore be required to determine whether these sites contribute to the condensate behaviour described under hyperosmotic conditions.

A further limitation is the limited coverage of the canonical WNK1 signaling pathway in the present phosphoproteomic datasets. Established WNK1-associated proteins, including OXSR1, STK39, SLC12A1, SLC12A2, SLC12A5, SLC12A6, KLHL3, CUL3, and WNK2–WNK4 were not detected in the analysed PsOP tables. In addition, the canonical WNK1 phosphosites S382 and S1261 were detected in only 5 and 27 of the 352 differential datasets, respectively, and neither site was carried forward for site-pair analysis. These observations should not be interpreted as evidence that these proteins or phosphosites are not associated with WNK1. Rather, they highlight the dependence of the present analysis on the coverage and composition of the available phosphoproteomic datasets. As an internal consistency observation, TSC22D4 was identified as a binary interactor and NRBP2 as a candidate kinase, consistent with previously reported WNK1- and NRBP-related biology.

Despite these inferences, the co-differential associations reported here provide statistical evidence of phosphosite co-occurrences across aggregated and heterogeneous phosphoproteomic datasets spanning multiple cell types, experimental conditions, and mass spectrometry platforms. The bias-correction filters (magnitude ratio, independent-study, and distinct-condition thresholds) were applied to reduce the influence of any single dataset or study; however, they do not establish a direct biochemical or causal relationship between WNK1 and any individual PsOP. Similarly, the upstream kinase and substrate predictions drawn from the Johnson et al. (2023) [43] kinase-substrate specificity atlas are sequence-based computational predictions applied to the three major phosphosites. They therefore represent candidate associations that require independent experimental validation, such as in vitro kinase assays, phosphosite mutagenesis, or targeted knockdown studies, before any of the proteins identified here can be considered validated WNK1 regulators, interactors, or substrates. Overall, the GO enrichment analysis indicates site-specific functional associations among proteins linked to WNK1 S2027, S2029, and S2032. S2027-associated proteins were primarily enriched in processes related to chromatin modification, Ras/small GTPase signaling, and cell-cycle regulation, whereas S2029-associated proteins showed stronger enrichment in chromosome organization and transcriptional regulatory functions. In contrast, S2032-associated proteins were particularly enriched in GTPase-related molecular functions, with no significant enrichment detected for biological processes. These differences suggest that the three WNK1 frequently detected phosphosites may be associated with distinct functional protein networks, although the GO analysis represents functional enrichment of associated proteins and does not establish direct mechanistic relationships.

Taken together, these findings provide a candidate hypothesis-generating map of phosphosite associations within a comparatively uncharacterised region of WNK1, rather than a validated signaling network. The associations involving the S2027/S2029/S2032 region, stress-responsive kinases, cytoskeletal regulators of migration, and the previously described IDR-related biology provide potential directions for targeted experimental follow-up. In particular, defined osmotic perturbation experiments and site-specific mutational studies will be needed to determine whether the identified phosphosites contribute directly to the osmotic and condensate-related properties reported for the WNK1 IDR. 

## 5. Conclusions

This study assembled a curated corpus of human phosphoproteomic data to characterise the co-differential association network surrounding the three most frequently detected phosphosites in the C-terminal intrinsically disordered region of WNK1, S2032, S2029 and S2027. Applying a stringent multi-filter statistical pipeline, we retained 633 WNK1 site-PsOP pairs. These comprise 387 distinct phosphosites in other proteins (PsOPs) with positive co-differential association, located on 324 proteins, and 34 distinct phosphosites with negative association, located on 34 proteins. Within this set, we identified potential upstream kinases, substrates and binary interactors. Gene Ontology enrichment of these PsOPs converged on themes of Ras/small GTPase signaling, chromatin organization, and the cell cycle.

Together, these results outline a candidate signaling map for a comparatively uncharacterised region of WNK1. This work should be treated as hypothesis-generating resource which requires future experimental investigations to validate these associations and determine their underlying molecular mechanisms and functional relevance.

## Data Availability

The original contributions presented in this study are included in the article and Supplementary Materials. Further inquiries can be directed to the corresponding author.

## Supplementary Materials

The following supporting information can be downloaded at https://www.mdpi.com/article/10.3390/biotech15040095/s1, Table S1: Human cellular WNK1 profile data; Table S2: Human cellular WNK1 differential data; Table S3: Differential frequency table of WNK1; Table S4: List of WNK1 S2027 positively co-differentially associated phosphosites in other proteins; Table S5: List of WNK1 S2027 negatively co-differentially associated phosphosites in other proteins; Table S6: List of WNK1 S2029 positively co-differentially associated phosphosites in other proteins; Table S7: List of WNK1 S2029 negatively co-differentially associated phosphosites in other proteins; Table S8: List of WNK1 S2032 positively co-differentially associated phosphosites in other proteins; Table S9: List of WNK1 S2032 negatively co- differentially associated phosphosites in other proteins; Table S10: Binary interactors of WNK1: S2027, S2029, S2032; Table S11: Upstream kinases of WNK1: S2027, S2029, S2032; Table S12: Additional kinases of WNK1: S2027, S2029, S2032; Table S13: Substrates of WNK1: S2027, S2029, S2032; Table S14: Background and input genes for Gene Ontology (GO) enrichment analysis; Table S15: Gene Ontology (GO) enrichment analysis of genes positively and negatively co-differentially associated with WNK1 frequently detected phosphosites.

## Abbreviations

The following abbreviations are used in this manuscript:

IDR	Intrinsically Disordered Region
PHAII	Pseudohypoaldosteronism Type II
PsOPs	Phosphosites in other proteins
FET	Fisher’s exact test
UUDD	Positive co-differential association
UDDU	Negative co-differential association
ASD	Autism spectrum disorder
PtdIns	Phosphatidylinositol
NPC	Neural progenitor cells
GEF	Guanine nucleotide exchange factor
EMT	Epithelial-to-mesenchymal transition
